# Epistasis analysis of microRNAs on pathological stages in colon cancer based on an Empirical Bayesian Elastic Net method

**DOI:** 10.1186/s12864-017-4130-7

**Published:** 2017-10-16

**Authors:** Jia Wen, Andrew Quitadamo, Benika Hall, Xinghua Shi

**Affiliations:** Department of Bioinformatics and Genomics, College of Computing and Informatics, University of North Carolina at Charlotte, Charlotte, NC 28223 USA

**Keywords:** Colon cancer, Empirical Bayesian Elastic net, Epistasis, microRNAs

## Abstract

**Background:**

Colon cancer is a leading cause of worldwide cancer death. It has become clear that microRNAs (miRNAs) play a role in the progress of colon cancer and understanding the effect of miRNAs on tumorigenesis could lead to better prognosis and improved treatment. However, most studies have focused on studying differentially expressed miRNAs between tumor and non-tumor samples or between stages in tumor tissue. Limited work has conducted to study the interactions or epistasis between miRNAs and how the epistasis brings about effect on tumor progression. In this study, we investigate the main and pair-wise epistatic effects of miRNAs on the pathological stages of colon cancer ﻿using datasets from The Cancer Genome Atlas.

**Results:**

We develop a workflow composed of multiple steps for feature selection based on the Empirical Bayesian Elastic Net (EBEN) method. First, we identify the main effects using a model with only main effect on the phenotype. Second, a corrected phenotype is calculated by removing the significant main effect from the original phenotype. Third, we select features with epistatic effect on the corrected phenotype. Finally, we run the full model with main and epistatic effects on the previously selected main and epistatic features. Using the multi-step workflow, we identify a set of miRNAs with main and epistatic effect on the pathological stages of colon cancer. Many of miRNAs with main effect on colon cancer have been previously reported to be associated with colon cancer, and the majority of the epistatic miRNAs share common target genes that could explain their epistasis effect on the pathological stages of colon cancer. We also find many of the target genes of detected miRNAs are associated with colon cancer. Go Ontology Enrichment Analysis of the experimentally validates targets of main and epistatic miRNAs, shows that these target genes are enriched for biological processes associated with cancer progression.

**Conclusion:**

Our results provide a set of candidate miRNAs associated with colon cancer progression that could have potential translational and therapeutic utility. Our analysis workflow offers a new opportunity to efficiently explore epistatic interactions among genetic and epigenetic factors that could be associated with human diseases. Furthermore, our workflow is flexible and can be applied to analyze the main and epistatic effect of various genetic and epigenetic factors on a wide range of phenotypes.

**Electronic supplementary material:**

The online version of this article (10.1186/s12864-017-4130-7) contains supplementary material, which is available to authorized users.

## Background

Colon cancer is the third most common cancer worldwide, and is the second leading cause of cancer deaths in Europe and the United States [1–3]. Both genetic and epigenetic alterations have been implicated in the development of colon cancer [4]. microRNAs (miRNAs) are small (18–24 nucleotides) noncoding RNAs, that act as epigenetic regulation of gene expression. miRNAs act on genes post-translationally and have been implicated in cancer development, progression, and both response and resistance to chemotherapy [5]. Alterations of miRNA expression have been detected in the broad spectrum of hematological malignancies and solid tumors, including colon cancer [6–10]. Previous studies have established that miRNAs are differentially expressed in tumor and normal tissue [5], and altered miRNA expression is involved in colon cancer development [6, 11, 12, 13, 14, 37]. For example, miR-144 is significantly associated with colon tumor stages [15]. Therefore, the expression changes of microRNAs may regulate important genes in tumor pathogenesis and can be useful for classifying tumors and predicting their outcomes.

However, most studies focus on the identifying differentially expressed miRNAs between tumor and non-tumor samples or between stages in tumor tissue. Limited work has conducted to study the interactions or epistasis between miRNAs and how the epistasis brings about effect on tumor progression. Here, we define epistasis as the situations that the phenotype variance could be explained by the interactions or combinations of (epi-)genetic variants, instead of individual (epi-)genetic variants alone. Epistasis of miRNAs have been reported as an important component in cancer research and drug resistance research. For example, a previous study has reported an epistasis between miR-155 and miR-146a related to tumor growth [16]. Specifically, this study identifies that miR-155 deficiency is epistatic to a loss of miR-146a during antitumor immune responses and thus results in varied tumor growth [16].

However, genomic data is usually high dimensional, making it difficult to analyze epistatic interactions using general parameter estimate methods, such as variations of LASSO [17–24] or the empirical Bayesian method [25]. Many other methods developed to analyze epistasis on quantitative phenotypes, including a statistical selection method [26] and a combinatorial partitioning method (CPM) for multi-locus-epistasis [27]. A multifactor-dimensionality reduction method (MDR) [28] is developed based on CPM, and a GEM model is developed to detect the functional epistasis and infer the hierarchical relationships of genes [29]. Neither CPM or MDR scales up well, so it is impractical to use them on large datasets [27, 28].

Additionally, methods have been proposed to identify epistasis on dichotomous phenotypes as in case-control studies. These methods include an Epistasis Detector based on the Clustering of relatively Frequent items (EDCF) [30], a Bayesian inference method called Detecting genome-wide Association on Multiple diseases (DAM) [31], a Multi-SNP Combination Set Detector (MSCD) based on a combinatorial optimization model [32].

Recently, an Empirical Bayesian Elastic Net (EBEN) method was proposed to study epistasis [33]. EBEN is efficient to estimate unknown parameters in an over- saturated statistical model as in mining high dimensional genomic data. Therefore, in this study, we use the EBEN method thanks to three of its advantages for epistasis analysis: 1) EBEN is scalable on high dimensional data, 2) EBEN can perform a statistical test on the features selected, and 3) EBEN shows lower FDR than LASSO [33].

In order to efficiently identify epistasis that might be masked by strong main effects, we develop a multi-step workflow to find both main and epistatic effect in a unified model. First, we identify the main effect of miRNAs on pathological stages of colon cancer, ignoring epistasis. Second, we generate a corrected phenotype by removing the main effect. Third, we use an epistasis model on the corrected phenotype to solely identify epistatic effect. Finally, we run a full model including both main and epistatic effects, on the significant features previously selected as main and epistatic effects. We apply EBEN as the parameter estimation method in all steps.

Using the multi-step workflow on data from the The Cancer Genome Atlas (TCGA) [34], we identify a set of miRNAs with main and epistatic effect on the pathological stages of colon cancer. Many of miRNAs with main effect we detected have been reported to be associated with colon cancer from previous experimental studies, and the majority of epistatic miRNAs share common target genes and thus could bring about epistatic effect on the resulted pathological stages. We also find some of the target genes of detected miRNAs are associated with colon cancer. Gene Ontology Enrichment Analysis of the experimentally validates targets of main and epistatic miRNAs, shows that these target genes are enriched for biological processes associated with cancer progression.

## Methods

In order to efficiently identify the main and epistatic effect of miRNAs on pathological stages, we develop a multi-step workflow based on the Empirical Bayesian Elastic Net method for modeling. We use the miRNA profiles and pathological stages of colon cancer as an example to demonstrate our analysis workflow. The data used in this study, R script pipeline on analyzing the dataset, and the EBEN package can be accessed from github (https://github.com/shilab/EBEN-epistasis) and R CRAN (https://cran.r-project.org/web/packages/EBEN/index.html).

### Data collection and preprocessing

TCGA [34] provides a dataset that fits well to evaluate the proposed method, since it offers comprehensive measurements at different layers on the same individuals for a cancer type for integrative analysis. The miRNA expression profiles from miRNA sequencing (miRNASeq) and clinical data for colon cancer were downloaded from TCGA data portal. Specifically, we extracted miRNA expression data and pathological stages of 233 samples from the TCGA colon cancer datasets. We then filter out those miRNAs with more than 20% missing data and finally collected the expression profiles of 376 miRNAs in 233 samples. We then organize the miRNA expression data into a matrix, with each row representing a sample and each column representing a miRNA. We use inverse quantile normalization on the miRNA expression matrix, map the values for each miRNA onto a standard normal distribution, and transpose our miRNA expression matrix for analysis.

In this study, we focus on analyzing the impact of individual miRNAs, and the epistasis between two miRNAs on the pathological stage of colon cancer. Here, we use the pathological stages, i.e., tumor stages, as a proxy to study cancer progression. According to TCGA, the pathological stage refers the “classification assigned to a malignancy which allows for the grouping of similar cancer types based on the extent of disease in the primary tumor (T), regional lymph nodes (N), and metastatic sites (M), using criteria from the American Joint Committee on Cancer staging criteria” [34]. We extracted the pathological stages of these 233 samples from TCGA. The pathological stages are then transformed into natural log values to scale the ordinary value of different pathological stages in order to make the variation more similar across different ordinary values.

### Introduction of empirical bayesian elastic net

In our workflow, we use an Empirical Bayesian Elastic Net (EBEN) to model the data. We choose EBEN because it scales well on high-dimensional data since it uses feature filtering to remove unimportant features and the coordinate ascent method to estimate the unknown parameters. The unknown parameters in the EBEN algorithms are *μ* , *β* and $\overset{\sim }{\alpha }$ in the linear model in Eq. (5). *μ* denotes the mean of phenotype that is assigned to a uniform prior distribution. *β* is the coefficient matrix in the model of Eq. (5), and is what we aim to estimate for feature selections. *β* is assigned to have two-level prior distributions, with the first level as an independent normal distribution and the second level as a generalized Gamma distribution. EBEN algorithm introduces two hyper-parameters, *λ*_1_ and *λ*_2_, and then uses cross-validation to determine the optimal values of these two hyper-parameters. $\overset{\sim }{\alpha }$ is defined as $1/{\overset{\sim }{\sigma}}^2$, and ${\overset{\sim }{\alpha}}_k$ denotes the element of $\overset{\sim }{\alpha }$. In each cycle of the coordinate ascent method, EBEN adds or deletes features according to the variable of ${\overset{\sim }{\alpha}}_k$ between two iterations in the algorithm. If ${\overset{\sim }{\alpha}}_k$ is finite, feature *k* is kept in the model, otherwise it is deleted from the model. We can see that if the dataset is high dimensional, lots of ${\overset{\sim }{\alpha}}_k$ might be infinite using a coordinate ascent method, hence their corresponding *β* is zero and EBEN can drop them from model quickly. Therefore, EBEN is efficient to estimate unknown parameters in an over-saturated statistical model [33], makes it scalable to handle high dimensional datasets. Another reason that we prefer EBEN over other Elastic Net or LASSO methods because other methods usually give non-zero coefficients for feature selection, without estimating the covariance or performing a statistical test. Instead, EBEN performs a *t*-test using the coefficient and the covariance matrix to obtain *p*-values for selected features from point estimates [33].

**Fig. 1:**
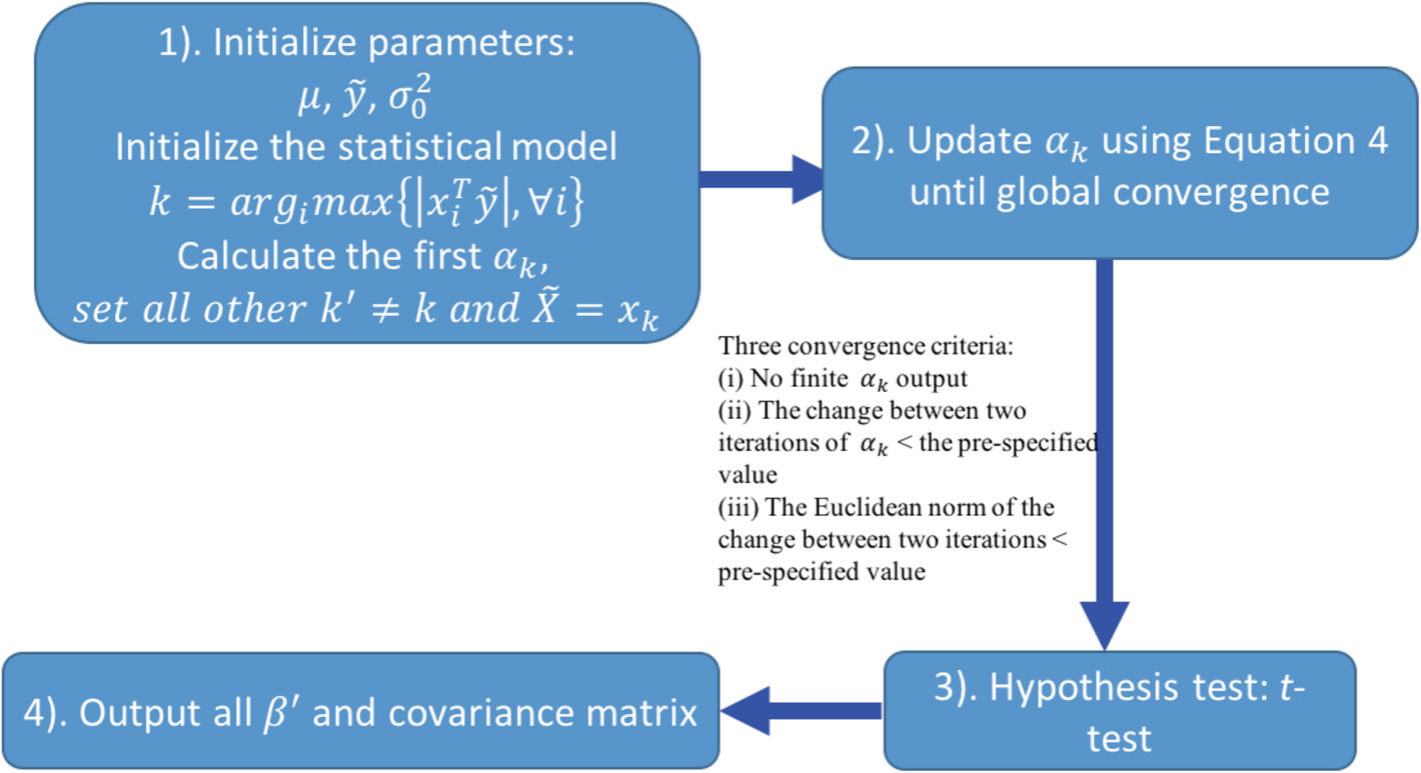
An overview of the EBEN algorithm. 1) Initialize model parameters and the statistical model. The unknown parameters *μ* denotes the mean of phenotype, $\overset{\sim }{y}$ denotes the initial dependent variable and ${\sigma}_0^2$ denotes the variance of the model, obtain the initial features satisfying $k={arg}_i\left\{\left|{x}_i^T\overset{\sim }{y}\right|,\forall i\right\}$. Here, *k* denotes the subscripts of features, *x*_*i*_ denotes the vector of feature *i*, $\overset{\sim }{y}$ denotes the dependent variable in the statistical model, and *α*_*k*_ is a variable calculated from ${\sigma}_k^2$, 2) Update the parameters in the model during iterations, 3) Use *t*-test to perform hypothesis test on the estimated value, and 4) Output *β* ^′^ that denotes the significant results and the covariance matrix

As illustrated in Fig. 1, the EBEN algorithm [33] can be summarized as the following four steps.

1. Initialize model parameters and the statistical model. The parameter sets need to be initialized are $\mu, {\sigma}_0^2$ and $\overset{\sim }{y}$. μ denotes the mean of phenotype and is initialized as $\mu =\frac{\sum_i^n{y}_{\mathrm{i}}}{n}$. $\overset{\sim }{y}$ denotes the initial dependent variable and is initialized as $\overset{\sim }{y}=y-\mu$. ${\sigma}_0^2$ denotes the variance of the model and can be initialized as a very small number such as ${\sigma}_0^2=0.1\times \frac{{\overset{\sim }{y}}^T\overset{\sim }{y}}{n}$ . After initializing these parameters, we need to initialize the statistical model with an initial set of features. The initial feature set satisfies $k={arg}_i\left\{\left|{x}_i^T\overset{\sim }{y}\right|,\forall i\right\}$, because EBEN starts with features that have the highest correlations with the dependent variable. Here, *n* is the number of samples, *k* denotes the subscripts of features, *x*_*i*_denotes the vector of feature *i*, and *α*_*k*_ is a variable calculated from ${\sigma}_k^2$.
2. For the posterior estimate, the posterior distribution of parameter set *θ* can be given as in Eq. (1) and the log posterior distribution of ${\overset{\sim }{\alpha}}_k$ in Eq. (2) according to the prior distributions [33]. The ${\overset{\sim }{\alpha}}_k$ is the element of $\overset{\sim }{\alpha }$, and *s*_*k*_ and *q*_*k*_ in Eq. (2) can be derived from *C* which is the covariance matrix of *y* calculated by the given $\overset{\sim }{\alpha }$ in Eq. (2) [33]:
  1 $$ p\left(\theta |y\right)\propto p\left(y|\mu, \beta, {\sigma}_0^2\right)p\left(\mu \right)p\left({\sigma}_0^2\right)p\left(\beta |{\overset{\sim }{\sigma}}^2\right)p\left({\overset{\sim }{\sigma}}^2|{\lambda}_1,{\lambda}_2\right) $$
  2 $$ L\left({\overset{\sim }{\alpha}}_k\right)=\frac{1}{2}\left[\mathit{\log}\frac{{\overset{\sim }{\alpha}}_k}{{\overset{\sim }{\alpha}}_k+1+{s}_k} + \frac{{\overset{\sim }{q}}_k^2}{{\overset{\sim }{\alpha}}_k+1+{s}_k\ }\right]-\frac{\lambda_2}{{\overset{\sim }{\alpha}}_k} $$

Let the $L\left({\overset{\sim }{\alpha}}_k\right)$ to be maximized, we can derive the optimal estimate of ${\overset{\sim }{\alpha}}_k$ as in Eq. (3) [33]:

3 $$ {\overset{\sim }{\alpha}}_k^{\ast }=\left\{\begin{array}{c}r, if\ {q}_k^2-{s}_k>{\lambda}_1+2{\lambda}_2\\ {}\infty, otherwise\end{array}\right. $$

*r* can be calculated according to the *s*_*k*_, *q*_*k*_, *λ*_1_ and *λ*_2_. From Eq. (3), the *β*_*k*_ will be zero if the ${\overset{\sim }{\alpha}}_k^{\ast }$ is infinite. During iterations, the algorithm finds a new *α*_*k*_ according to Eq. (4) [35]:

4 $$ j={arg}_k\left\{\varDelta L\left({\alpha}_k^{\ast}\right)=L\left({\alpha}_k^{\ast}\right)-L\left({\alpha}_k^{(n)}\right)\right\} $$

The parameters in the model are updated through iterations until three convergence criteria are met. These three criteria are i) no finite *α*_*k*_ is output, ii) the change between two iterations of *α*_*k*_ is smaller than a pre-specified value and iii) the Euclidean norm of the change between two iterations is smaller than a pre-specified value. There are two hyper-parameters in the algorithm, and EBEN uses cross-validation to determine the optimal value of hyper-parameters [33, 35].

3. Use the non-zero coefficients *β* and covariance matrix to conduct *t*-test to perform hypothesis test on the estimated value.
4. Output final *β* ^′^ that denotes the significant results and the covariance matrix.

### Our analysis workflow based on EBEN

In this study, we use a linear regression model to model the natural log value of pathological stages versus the main and pair-wise epistasis of miRNAs, and used the following formula as our full model (5):

5 $$ y=\mu +X{\beta}_m+{X}_i{X}_j{\beta}_e+e $$

where *y* denotes the dependent variable, (i.e., the transformed value using natural log on pathological stage in this study), (5) denotes the mean of miRNA expression level, *X* is the miRNA expression matrix with the dimension *n* × *k*, *n* is the sample size, *k* is the number of miRNAs, *β*_*m*_ is the coefficient that represents the main effect of miRNA, *X*_*i*_ and *X*_*j*_ denote two different miRNAs expression vectors, *β*_*e*_ is the coefficient that represents the epistasis between miRNA *i* and *j*, and *e* is the residual error that follows a normal distribution with zero mean and variance of *σ* ^2^, *e* ~ *N*(0, *σ* ^2^). Because EBEN could give the estimates of posterior variances, *t*-test was used to determine whether the non-zero coefficients of select features were significant.

**Fig. 2:**
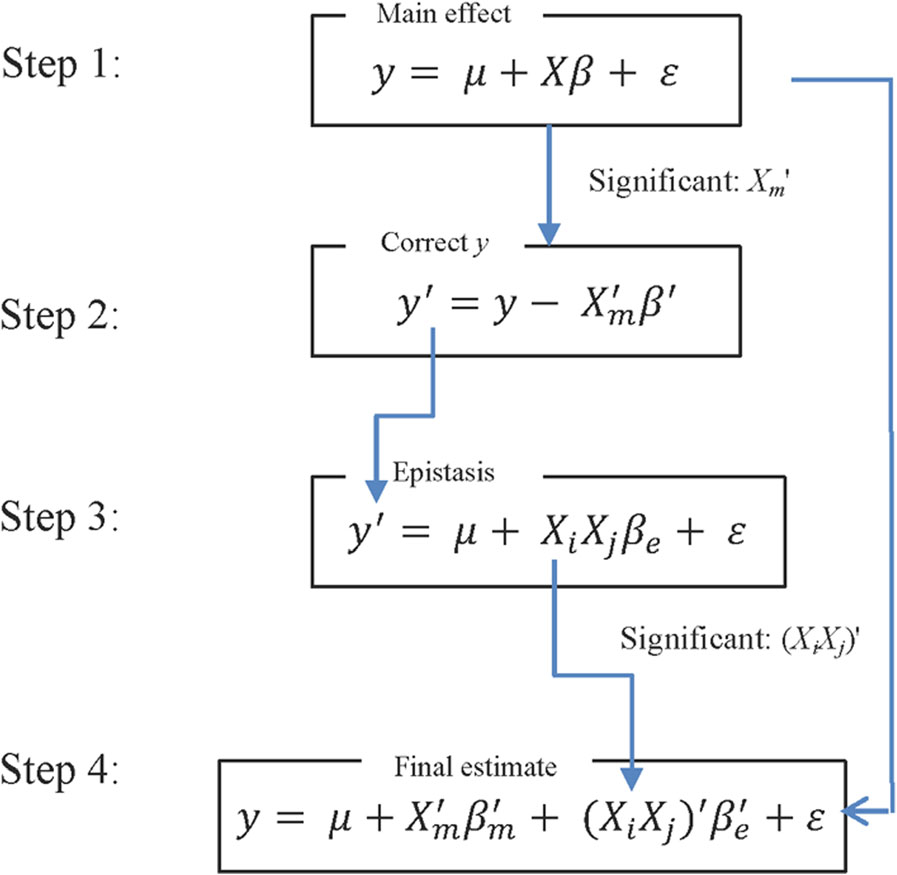
The overall workflow of our epistasis analysis based on EBEN. Step 1: Run the simplified model including solely main effect, which means only the significant features from this step are included in the main effect part of the model at the final step, Step 2: Obtain the corrected phenotype *y* ^′^ through removing main effect of significant features from the original phenotype *y*, Step 3: The newly corrected *y* ^′^ is used to infer epistasis. Only the features with significant epistatic effect can be included in the model in the next step. Step 4: Run the full model that includes all the features with significant features with main effects from the first step and significant features with epistatic effect identified in third step. EBEN is used as the parameter estimation method in Steps 1, 3 and 4. Here, *y* denotes the trait phenotype, *X* represents the miRNA expression, *β*_*m*_ and *β*_*e*_ represents for the main effect and epistatic effect separately, *μ* represents for the phenotype mean and e represents for the standard error

In order to avoid the situations that main effects dominate and mask out epistatic effects, we develop an analysis workflow composed of multiple steps of feature se- lection and modeling using BEN. The overall analysis workflow is illustrated in Fig. 2, and can be divided into the following four steps.

- Step 1: Select features with solely main effect *Xβ*_*m*_ on the phenotype *y*. EBEN was used to screen all the main features that have *p* values smaller than 0.05. Only these significant features with main effect would be included in the model of Step 4.
- Step 2: Derive a corrected phenotype with main effects removed. We eliminated those main effects from the original phenotype ($\overrightarrow{y}$) using the significant features with main effect from Step 1, to generate corrected *y* ^′^, ${y}^{\prime }=\overrightarrow{y}-{X}_m^{\prime }{\beta}_m^{\prime }$. ${X}_m^{\prime }$ represents the significant features selected in Step 1 and ${\beta}_m^{\prime }$ is the vector effects for the significant features ${X}_m^{\prime }$.
- Step 3: Select features with epistatic effect on the phenotype. The corrected *y* ^′^ was used as the new dependent variable to detect epistasis using EBEN. The significance epistatic effects were still selected at *p* level of 0.05.
- Step 4: A unified model of estimating both main and epistatic effect. All the features with main effect from the step 1 and epistatic features identified in Step 3 were included in Eq. 5 and estimated by EBEN. In this step, since the covariance matrix only included the significant main and epistasis effects from Steps 1 and 3, the new *p* values, *β*_*m*_ and *β*_*e*_ are different from the results in Steps 1 and 3. In order to obtain these values from the same model, we should use the new covariance matrix to re-estimate all the features to see whether each of them was significantly associated with phenotype. Here, the threshold value was also set at the level of *P* < 0.05.

## Results

Using our multi-step analysis workflow, we identify a set of miRNAs with main effect and epistatic effect, as summarized in Table 1. Many of miRNAs with main effect are verified to be up or down regulated in colon cancer by previous experimental studies (Table 2), and the majority pairs of epistatic miRNAs have common target genes that are associated with colon cancer. The target genes of these miRNAs related to pathological stages of colon cancer are previously reported to be associated with colon cancer. Further, we use the experimentally validated target genes of these identified miRNAs to conduct GO Enrichment Analysis, and find that these genes are enriched for biological processes related with cancer.

**Table 1:** The main and epistatic effect miRNAs identified to be associated with pathological stages of colon cancer in our study

Effect-type	miRNAs	*β*
Main Effect	hsa-let-7c	−0.0321
	hsa-mir-1249	−0.0668
	hsa-mir-31	0.0466
	hsa-mir-3189	−0.0475
	hsa-mir-320c-1	−0.0535
	hsa-mir-337	−0.0633
	hsa-mir-34a	−0.0382
	hsa-mir-3662	−0.0630
	hsa-mir-548e	−0.0404
	hsa-mir-580	0.0400
	hsa-mir-3065	0.0512
Epistatic Effect	hsa-let-7d, hsa-mir-548v	−0.0077
	hsa-mir-1254, hsa-mir-3615	0.0559
	hsa-mir-223, hsa-mir-3913-1	−0.0126
	hsa-mir-296, hsa-mir-432	0.0073
	hsa-mir-3131, hsa-mir-874	0.0078
	hsa-mir-3150b, hsa-mir-3610	0.0512
	hsa-mir-363, hsa-mir-937	0.0090
	hsa-mir-3682, hsa-mir-483	0.0020
	hsa-mir-3917, hsa-mir-3928	−0.0462
	hsa-mir-433, hsa-mir-616	−0.0383
	hsa-mir-496, hsa-mir-937	0.0143
	hsa-mir-511-1, hsa-mir-7-2	0.0069
	hsa-mir-3065, hsa-mir-656	0.0284
	hsa-mir-577, hsa-mir-92b	−0.0189

The effect-type describes either main or epistatic effect. miRNAs denote either individual miRNAs with main effect or miRNA pairs with epistatic effect. *β* values describe the effect sizes of selected miRNAs or miRNA pairs, learned from the model

**Table 2:** Our identified miRNAs with main effect that are previously reported to be associated with colon cancer

miRNA	up/down regulated	Verification	Reference
hsa-let-7c	down	qPCR	[43]
hsa-mir-1249	up	Microarray	[58]
hsa-mir-31	up	Northern Blot, qPCR	[37]
hsa-mir-31	up	Northern Blot, qPCR	[6, 43]
hsa-mir-31	up	Microarray	[59, 60]
hsa-mir-320c-1	down	qPCR	[43]
hsa-mir-337	up	Elastic-net regression, Microarray	[61, 62]
hsa-mir-34a	down	Microarray, Northern Blot	[59]
hsa-mir-34a	up	Northern Blot, qPCR	[13, 63]

Regarding the computational cost, the analysis takes approximately 20 h on a computing node with 2GB memory per process for the dataset consisting 376 features and 233 samples in this study. Most of the computing time is spent in training the model using cross-validation to choose the optimal hyperparameters. Once a model is learned and these hyperparameters are determined, it takes only tens of minutes to run the model on a dataset at this scale.

### Main effect

We identify 11 miRNAs with main effect on pathological stages of colon cancer, with 6 miRNAs being verified to be associated with colon cancer in previous experimental studies (Table 2). Table 2 summarizes the main effect miRNAs identified in our study that have been previously reported to be associated with colon cancer. For example, hsa-let-7c has been found to have an effect on regulating *RAS* oncogene expression in human colon cancer and hsa-let-7c could be involved in the growth of colon cancer cells [36]. In addition, miR-31 has the positive correlation with tumor stage in colon cancer [37]. Quantitative real-time PCR experiments find that miR-31 has the most notable oncogenic targets *AXIN1*, which is involved in Wnt signaling pathway and forkhead family transcription factors *FOXC2* and *FOXP3*, and this target gene and the two transcription factors are correlated with tumor stages [6]. Another example is that hsa-mir-1249 is found in our study, and *TP53* is one of hsa-mir-1249 target genes which is tumor protein gene. Mutations in *TP53* are one of the frequent alterations in human cancers. *TP53* is associated with poor prognosis in colon cancer and usually mutated in stage IV. *TP53* mutations have also be used as biomarkers in clinical settings [38].

### Epistatic effect

**Fig. 3:**
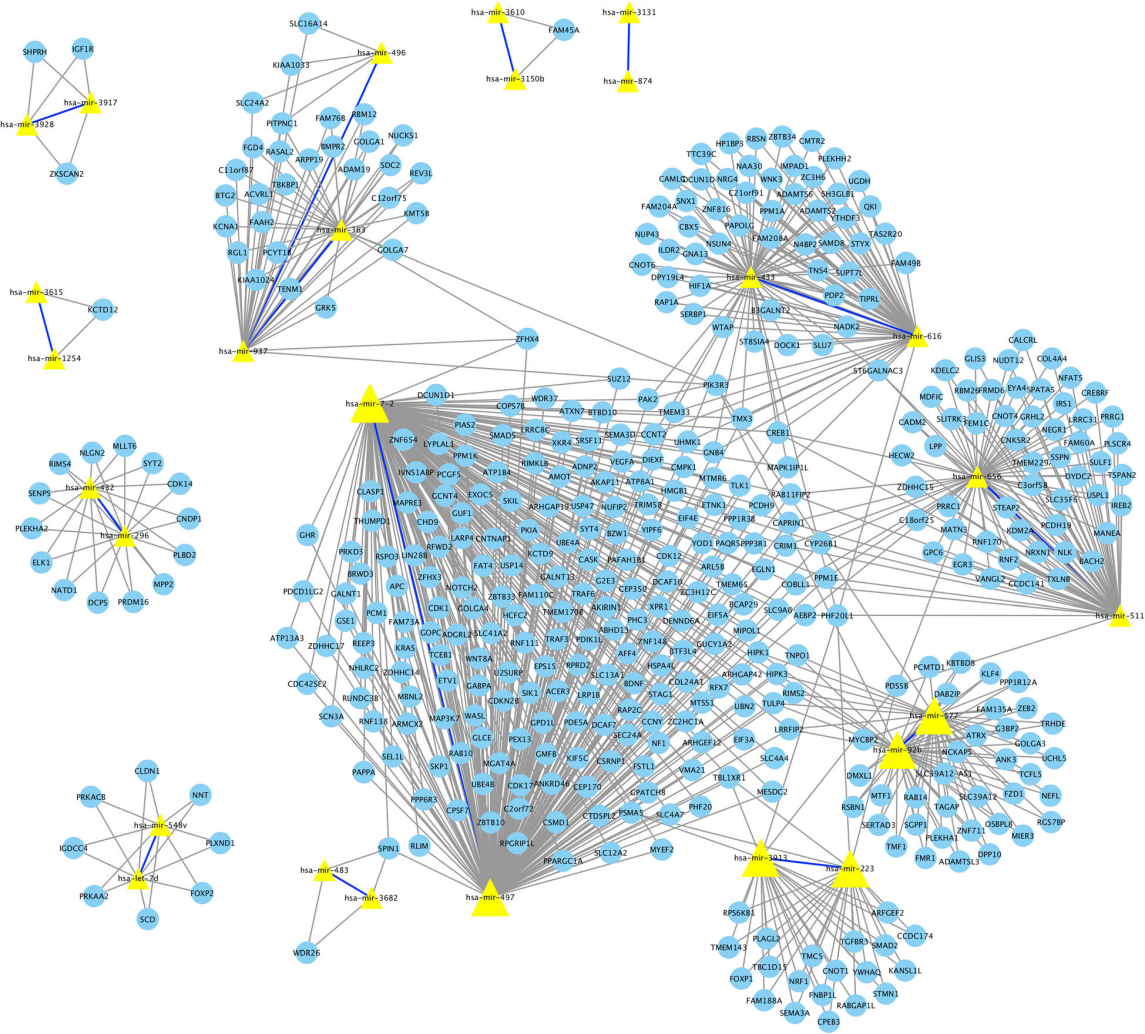
Common target genes shared by epistatic miRNAs. The target genes in this network are from TargetScan, miR2DB and miRDisease. The miRNAs are denoted by yellow triangles. Identified epistatic interactions between miRNAs are showed by solid bold blue lines. Common target genes of these epistatic miRNAs are denoted by blue circles. The common target genes with corresponding miRNAs are linked by solid black lines

For the epistasis analysis, we identify 14 pairs of epistatic miRNAs associated with pathological stage in colon cancer. 13 pairs among them have more than one common target genes according to three databases that are miR2Disease [39], TargetScan [40] and miRDB [41]. Figure 3 presents a network view among the epistatic miRNAs and their target genes. In Fig. 3, solid bold blue lines denote the epistasis between miRNAs, yellow triangles denote the miRNAs, their corresponding target genes are denoted as the blue dots, and the links between miRNAs and target genes are denoted by solid black lines. From this network, we can see that many epistatic miRNAs share the same target genes, which implies that the epistatic effect among miRNAs can be reflected by their joint effect on these common genes and potentially on the same pathways.

For instance, we find that hsa-mir-497 and hsa-mir-7-2 have an epistatic interaction and they share two common target genes, namely *APC* and *KRAS*. These two target genes belong to the Wnt signaling pathway and EGFR signaling pathway separately, which are involved in the development of colon cancer [42, 43]. Particularly, hsa-mir-7-2 is verified to negatively regulate the target *KLF4* and promotes the progress of colon cancer since *KLF4* is a tumor suppressor gene [44]. Recently, hsa-mir497 has been reported to have lower expression levels and be associated with progression in colorectal cancer [45]. Furthermore, *KLF4* is common target gene of both hsa-mir-497 and hsa-mir-7-2. Hence, we infer that hsa-mir-497 and hsa-mir-7-2 can affect the progression of colon cancer jointly in addition to their independent effect on colon cancer.

### Disease associations

In order to understand the associations between our target genes and colon cancer, we query our target genes of all miRNAs with both main and epistatic effect with Online Mendelian Inheritance in Man (OMIM) Disease database [46]. There are 26 genes associated with colon cancer reported in OMIM phenotype-gene relationships. Out of these 26 colon cancer related genes, we find that 15 genes are the target genes of the identified miRNAs associated with the pathological stages of colon cancer (Table 3). For example, *PIK3CA* is the target gene of hsa-mir-363, and a previous study [47] reports high frequency mutations of *PIK3CA* in colon cancer. *NRAS* is a target gene of six miNRAs that hsa-let-7c, hsa-let-7d, hsa-mir-363, hsa-mir-3913, hsa-mir-483 and hsa-mir-874 identified in our study. Another study [48] finds that the mutation of *NRAS* is related to the suppression of apoptosis in tumor development. *APC* is a target gene of four miRNAs that hsa-mir-22, hsa-mir-3065, hsa-mir-497 and hsa-mir-7-2 identified in our study, the mutation of *APC* can induce inherited syndromes familiar adenomatous polyposis which leads to a greater potential of colon cancer [49]. Additionally, *PIK3CA*, *NRAS* and *APC* are included in the most frequently mutated genes in colon cancer according to [50]. Hence, we propose that the main and epistatic relationship between miRNAs and colon cancer can be used as an evidence that these miRNAs might affect the prognosis and patient’s survival and can be used as biomarker future research of colon cancer.

**Table 3:** Summary of target genes associated with colon cancer according to OMIM database

Location	Phenotype	miRNA ID	Target genes
1p13.2	Colon cancer, somatic	hsa-let-7c, hsa-let-7d, hsa-mir-363,	NRAS
		hsa-mir-3913, hsa-mir-483, hsa-mir-874	
3q26.32	Colon cancer, somatic	hsa-mir-363	PIK3CA
4p16.3	Colon cancer, somatic	hsa-mir-296, hsa-mir-337, hsa-mir-874	FGFR3
5q22.2	Colon cancer, somatic	hsa-mir-22, hsa-mir-3065, hsa-mir-497, hsa-mir-7-2	APC
5q22.2	Colon cancer, somatic	hsa-mir-1249, hsa-mir-3662, hsa-mir-548e	MCC
		hsa-mir-548v, hsa-mir-7-2	
7q11.23	Colon cancer, somatic	hsa-mir-874	PTPN12
11p11.2	Colon cancer, somatic	hsa-mir-363, hsa-mir-497	PTPRJ
11q13.3	Colon cancer, susceptibility to	hsa-mir-432, hsa-mir-497, hsa-mir-511	CCND1
14q24.3	Colorectal cancer, somatic	hsa-mir-432	MLH3
14q32.33	Colorectal cancer, somatic	hsa-mir-1249, hsa-mir-548e, hsa-mir-656	AKT1
17p13.1	Colon cancer	hsa-mir-1249	TP53
17q24.1	Colorectal cancer, somatic	hsa-mir-1249, hsa-mir-497, hsa-mir-616	AXIN2
18q21.2	Colorectal cancer, somatic	hsa-mir-363, hsa-mir-3662, hsa-mir-3913, hsa-mir-548e	DCC
20q13.2	Colon cancer, susceptibility to	hsa-mir-363	AURKA
22q13.2	Colorectal cancer, somatic	hsa-let-7c, hsa-mir-497	EP300

## Gene ontology enrichment analysis

For the miRNAs identified as with main and epistatic effect on the pathological stages of colon cancer, we obtain all their experimentally verified target genes from miR2Disease. We then use these target genes from mir2Disease to conduct gene ontology (GO) enrichment analysis [51–53] to find out their enriched molecular functions and biological processes.

**Fig. 4:**
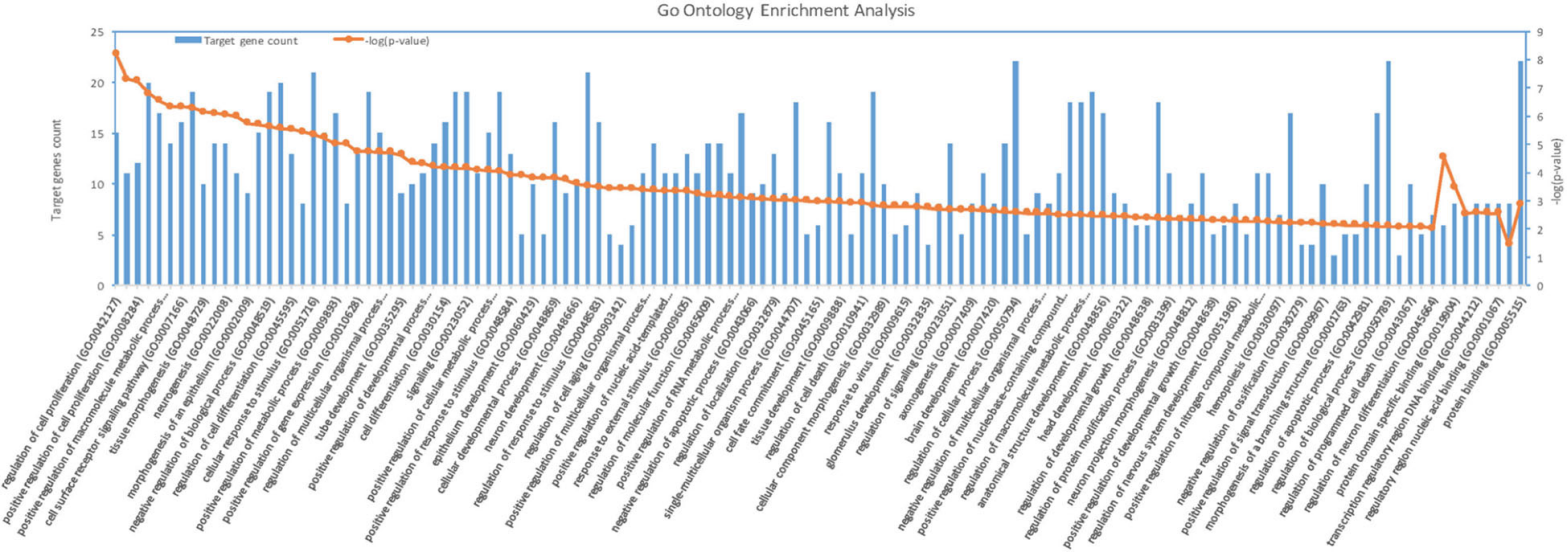
Gene Ontology Enrichment Analysis. X-axis represents each category of molecular functions and biological processes. Y-axis on the left denotes the target gene counts for each category and y-axis on the right denotes –log (*p*-value) values

As shown in Fig. 4, these target genes are enriched in biological processes including cell proliferation, cell death and cell division (see Additional file 1). Cell proliferation and cell death are related with tumors, and cell division is proved to be related with colon cancer [54]. Because the growth of tumor depends on the combined regulation of cell proliferation, cell death and cell division, cancer progression is possible if cell death is suppressed, and cell division and proliferation is promoted [54, 55]. These target genes are also enriched in molecular function including protein binding and regulatory region DNA binding. Studies have shown that protein binding and regulatory region DNA binding processes are associated with colon cancer [56, 57]. Thus, these target genes may serve as drug targets as they could block the progression of colon cancer by interfering with protein binding or regulation of DNA binding.

## Discussion

For the optimization problem in EBEN, we can improve its performance by setting initial random seed or increasing the fold number in cross validation of EBEN. We will further incorporate covariates, such as known and unknown confounders to EBEN method. Comparing with the nature log value to transform an ordinary phenotype, we will extend EBEN to directly take ordinary phenotypes. We can also make minor adjustments for ﻿different datasets, such as adjust the *p* value threshold in the step 1 and 3, in our pipleine used in this study.

While we use miRNAs in this analysis, we acknowledge that gene expression also plays an important role in colon cancer and leading to changes of tumor pathological stages. We plan to incorporate gene expression into the model that would allow us to study interactions between miRNAs and genes in a unified statistical model. We believe this strategy can help develop a better understanding of the molecular mechanisms of colon cancer. In addition, germline genetic variation and somatic genetic aberrations contribute significantly to tumorigenesis. Therefore, we plan to include these genetic factors into our model as well.

## Conclusion

Changes in miRNA expression are known to be involved in colon cancer development and progression. miRNAs have a critical impact on etiology of cancer and cancer progression. Clarifying the changes and the epistatic effect among miRNAs could be helpful to advance cancer research and treatment.

In this study, we apply a multi-step workflow that enables us to identify both main and pair-wise epistatic effects of miRNAs on pathological stages of colon cancer. In each learning step of the workflow, an Empirical Bayesian Elastic Net method is used to solve the model. It has been demonstrated that EBEN efficiently selects significant features in high dimensional (epi-)genomic datasets. While we use miRNA expression data and pathological stages in our study, this workflow can be used to identify epistasis and main effect in many diseases.

In summary, our study provides a flexible workflow for an integrative analysis of the contribution of genetic and epigenetic factors to phenotypes. Such analysis has potentials for biomarker and drug discovery, as well as for the improvement in prognosis prediction. Our study thus provides a reference pipeline for epistasis and main effect analysis in future research that can be extended to various applications.

## Additional file

Additional file 1: Table S1. Gene Ontology Enrichment Analysis. (PDF 69 kb)

## Abbreviations

CPM: Combinatorial partitioning method
DAM: Detecting genome-wide association on multiple diseases
EBEN: Empirical bayesian elastic net
EDCF: Clustering of relatively frequent items
GO: Gene ontology
MDR: Multifactor-dimensionality reduction method
miRNAs: microRNAs
MSCD: Multi-SNP combination set detector
OMIM: Online Mendelian inheritance in man
TCGA: The cancer genome atlas

## References

[1] Ferlay J, Autier P, Boniol M, Heanue M, Colombet M, Boyle P (2007). Estimates of the cancer incidence and mortality in Europe in 2006. Ann Oncol.

[2] Siegel R, Ward E, Hao Y, Xu J, Murray T, Thun MJ (2008). Cancer statistics, 2008. CA Cancer J Clin.

[3] Zhou JJ, Zheng S, Sun LF, Zheng L (2014). MicroRNA regulation network in colorectal cancer metastasis. World J Biol Chem.

[4] Sarver AL, French AJ, Borralho PM, Thayanithy V, Oberg AL, Silverstein KA (2009). Human colon cancer profiles show differential microRNA expression depending on mismatch repair status and are characteristic of undifferentiated proliferative states. BMC Cancer.

[5] Volinia S, Calin GA, Liu CG, Ambs S, Cimmino A, Petrocca F (2006). A microRNA expression signature of human solid tumors defines cancer gene targets. Proc Natl Acad Sci U S A.

[6] Slaby O, Svoboda M, Fabian P, Smerdova T, Knoflickova D, Bednarikova M (2007). Altered expression of miR-21, miR-31, miR-143 and miR-145 is related to clinicopathologic features of colorectal cancer. Oncology.

[7] Winter J, Jung S, Keller S, Gregory RI, Diederichs S (2009). Many roads to maturity: microRNA biogenesis pathways and their regulation. Nat Cell Biol.

[8] Esquela-Kerscher A, Slack FJ (2006). Oncomirs-microRNAs with a role in cancer. Nat Rev Cancer.

[9] Garzon R, Fabbri M, Cimmino A, Calin GA, Croce CM (2006). MicroRNA expression and function in cancer. Trends Mol Med.

[10] Garzon R, Calin GA, Croce CM (2009). MicroRNAs in cancer. Annu Rev Med.

[11] Michael MZ, O’Connor SM, van Holst Pellekaan NG, Young GP, James RJ (2003). Reduced accumulation of specific MicroRNAs in colorectal neoplasia. Mol Cancer Res.

[12] Croce CM, Calin GA (2005). miRNAs, cancer, and stem cell division. Cell.

[13] Schetter AJ, Leung SY, Sohn JJ, Zanetti KA, Bowman ED (2008). MicroRNA expression profiles associated with prognosis and therapeutic outcome in colon adenocarcinoma. JAMA.

[14] Schepeler T, Reinert JT, Ostenfeld MS, Christensen LL, Silahtaroglu AN, Dyrskjøt L (2008). Diagnostic and prognostic microRNAs in stage II colon cancer. Cancer Res.

[15] Kalimutho M, Blanco GD, Di Cecilia S, Sileri P, Cretella M, Pallone F (2011). Differential expression of miR-144* as a novel fecal-based diagnostic marker for colorectal cancer. J Gastroenterol.

[16] Huffaker TB, Hu R, Runtsch MC, Bake E, Chen X, Zhao J (2012). Epistasis between microRNAs 155 and 146a during T cell-mediated antitumor immunity. Cell Rep.

[17] Tibshirani R. Regression shrinkage and selection via the lasso**.** Journal of the Royal Statistical Society. Series B (Methodological). 1996;58(1):267–288.

[18] Quitadamo A, Tian L, Hall B, Shi X. An integrated network of microRNA and gene expression in ovarian cancer. BMC Bioinformatics. 2015;16(5):S5. doi:10.1186/1471-2105-16-S5-S5. 10.1186/1471-2105-16-S5-S5 PMC4402579 25860109

[19] Wang Z, Xu J, Shi X (2014). Finding alternative expression quantitative trait loci by exploring sparse model space. J Comput Biol.

[20] Tian L, Quitadamo A, Lin F, Shi X (2014). Methods for population-based eQTL analysis in human genetics. Tsinghua Sci Technol.

[21] Chen X, Shi X, Xu X, Wang Z, Mills R, Lee C (2012). A two-graph guided multi-task lasso approach for eQTL mapping. International Conference on Articial Intelligence and Statistics.

[22] Cheng W, Shi Y, Zhang X, Wang W (2015). Fast and robust group-wise eQTL mapping using sparse graphical models. BMC Bioinformatics.

[23] Cheng W, Zhang X, Guo Z, Shi Y, Wang W (2014). Graph-regularized dual Lasso for robust eQTL mapping. Bioinformatics.

[24] Kim S, Xing EP. Tree-guided group lasso for multi-response regression with structured sparsity, with an application to eQTL mapping. Ann Appl Stat. 2012:1095–117.

[25] Xu S (2007). An empirical Bayes method for estimating epistatic effects of quantitative trait loci. Biometrics.

[26] Huang Y, Wuchty S, Przytycka TM (2013). eQTL epistasis challenges and computational approaches. Front Genet.

[27] Nelson MR, Kardia SL, Ferrell RE, Sing CF (2001). A combinatorial partitioning method to identify multilocus genotypic partitions that predict quantitative trait variation. Genome Res.

[28] Ritchie MD, Hahn LW, Roodi N, Bailey LR, Dupont WD, Parl FF (2001). Multifactor-dimensionality reduction reveals high-order interactions among estrogen-metabolism genes in sporadic breast cancer. Am J Hum Genet.

[29] Kang M, Zhang C, Chun HW, Ding C, Liu C, Gao J (2015). eQTL epistasis: detecting epistatic effects and inferring hierarchical relationships of genes in biological pathways. Bioinformatics.

[30] Xie M, Li J, Jiang T (2012). Detecting genome-wide epistasis based on the clustering of relatively frequent items. Bioinformatics.

[31] Guo X, Zhang J, Cai Z, Du DZ, Pan Y. Searching genome-wide multi-locus associations for multiple diseases based on Bayesian Inference. IEEE/ACM Trans Comput Biol Bioinform. 2016;14(3):600–610. 10.1109/TCBB.2016.2527648 26887006

[32] Ding X, Wang J, Zelikovsky A, Guo X, Xie M, Pan Y (2015). Searching high-order SNP combinations for complex diseases based on energy distribution difference. IEEE/ACM Trans Comput Biol Bioinform.

[33] Huang A, Xu S, Cai X (2015). Empirical Bayesian elastic net for multiple quantitative trait locus mapping. Heredity.

[34] The Cancer Genome Atlas Project: The Cancer Genome Atlas (TCGA). https://tcga-data.nci.nih.gov/.

[35] Cai X, Huang A, Xu S (2011). Fast empirical Bayesian LASSO for multiple quantitative trait locus mapping. BMC Bioinformatics.

[36] Akao Y, Nakagawa Y, Naoe T (2006). Let-7 microRNA functions as a potential growth suppressor in human colon cancer cells. Biol Pharm Bull.

[37] Bandrés E, Cubedo E, Agirre X, Malumbres R, Zarate R, Ramirez N (2006). Identification by real-time PCR of 13 mature microRNAs differentially expressed in colorectal cancer and non-tumoral tissues. Mol Cancer.

[38] Olivier M, Hollstein M, Hainaut P (2010). TP53 mutations in human cancers: origins, consequences, and clinical use. Cold Spring Harb Perspect Biol.

[39] Jiang Q, Wang Y, Hao Y, Juan L, Teng M, Zhang X (2009). miR2Disease: a manually curated database for microRNA deregulation in human disease. Nucleic Acids Res.

[40] Lewis BP, Burge CB, Bartel DP (2005). Conserved seed pairing, often flanked by adenosines, indicates that thousands of human genes are microRNA targets. Cell.

[41] Wong N, Wang X. miRDB: an online resource for microRNA target prediction and functional annotations. Nucleic Acids Res. 2015:43(D1):D146–D152. 10.1093/nar/gku1104 PMC4383922 25378301

[42] Anastas JN, Moon RT (2013). WNT signaling pathways as therapeutic targets in cancer. Nat Rev Cancer.

[43] Vishnubalaji R, Hamam R, Abdulla MH, Mohammed MA, Kassem M, Al-Obeed O (2015). Genome-wide mRNA and miRNA expression profiling reveal multiple regulatory networks in colorectal cancer. Cell Death Dis.

[44] Meza-Sosa KF, Pérez-García EI, Camacho-Concha N, López-Gutiérrez O, Pedraza-Alva G, Pérez-Martínez L (2014). MiR-7 promotes epithelial cell transformation by targeting the tumor suppressor KLF4. PLoS One.

[45] Aherne ST, Madden SF, Hughes DJ, Pardini B, Naccarati A, Levy M (2015). Circulating miRNAs miR-34a and miR-150 associated with colorectal cancer progression. BMC Cancer.

[46] Online Mendelian Inheritance in Man (OMIM). http://omim.org/. Accessed May 2016.

[47] Samuels Y, Wang Z, Bardelli A, Silliman N, Ptak J, Szabo S (2004). High frequency of mutations of the PIK3CA gene in human cancers. Science.

[48] Haigis KM, Kendall KR, Wang Y, Cheung A, Haigis MC, Glickman JN (2008). Differential effects of oncogenic K-Ras and N-Ras on proliferation, differentiation and tumor progression in the colon. Nat Genet.

[49] Charames GS, Ramyar L, Mitri A, Berk T, Cheng H, Jung J (2008). A large novel deletion in the APC promoter region causes gene silencing and leads to classical familial adenomatous polyposis in a manitoba mennonite kindred. Hum Genet.

[50] Chisanga D, Keerthikumar S, Pathan M, Ariyaratne D, Kalra H, Boukouris S (2016). Colorectal cancer atlas: an integrative resource for genomic and proteomic annotations from colorectal cancer cell lines and tissues. Nucleic Acids Res.

[51] Carbon S, Ireland A, Mungall CJ, Shu S, Marshall B, Lewis S (2009). AmiGO: online access to ontology and annotation data. Bioinformatics.

[52] Ashburner M, Ball CA, Blake JA, Botstein D, Butler H, Cherry JM (2000). Gene Ontology: tool for the unification of biology. Nat Genet.

[53] Gene Ontology Consortium (2015). Gene ontology consortium: going forward. Nucleic Acids Res.

[54] Emmert-Streib F, de Matos Simoes R, Glazko G, McDade S, Haibe-Kains B, Holzinger A, et al. Functional and genetic analysis of the colon cancer network. BMC Bioinformatics. 2014, 15(6): Suppl 6. 10.1186/1471-2105-15-S6-S6 PMC4158620 25079297

[55] Ettarh R, Cullen A, Calamai A (2010). NSAIDs and cell proliferation in colorectal cancer. Pharmaceuticals.

[56] Li XL, Zhou J, Chen ZR, Chng WJ (2015). P53 mutations in colorectal cancer-molecular pathogenesis and pharmacological reactivation. World J Gastroenterol.

[57] Wang TY, Jia YL, Zhang X, Sun QL, Li YC, Zhang JH, et al. Treating colon cancer cells with FK228 reveals a link between histone lysine acetylation and extensive changes in the cellular proteome. Sci Rep. 2015;5 10.1038/srep18443 PMC4682073 26675280

[58] Li E, Ji P, Ouyang N, Zhang Y, Wang XY, Rubin DC (2014). Differential expression of miRNAs in colon cancer between African and Caucasian Americans: implications for cancer racial health disparities. Int J Oncol.

[59] Arndt GM, Dossey L, Cullen LM, Lai A, Druker R, Eisbacher M (2009). Characterization of global microRNA expression reveals oncogenic potential of miR-145 in metastatic colorectal cancer. BMC Cancer.

[60] Motoyama K, Inoue H, Takatsuno Y, Tanaka F, Mimori K, Uetake H (2009). Over-and under-expressed microRNAs in human colorectal cancer. Int J Oncol.

[61] Lee H, Flaherty P, Ji HP (2013). Systematic genomic identification of colorectal cancer genes delineating advanced from early clinical stage and metastasis. BMC Med Genet.

[62] Nishida N, Nagahara M, Sato T, Mimori K, Sudo T, Tanaka F, et al. Microarray analysis of colorectal cancer stromal tissue reveals upregulation of two oncogenic miRNA clusters. Clin Cancer Res. 2012;18(11):3054–70. 10.1158/1078-0432.CCR-11-1078 22452939

[63] Tazawa H, Tsuchiya N, Izumiya M, Nakagama H (2007). Tumor-suppressive miR-34a induces senescence-like growth arrest through modulation of the E2F pathway in human colon cancer cells. Proc Natl Acad Sci.

