# Supplementary material for: Epistasis analysis of microRNAs on pathological stages in colon cancer based on an Empirical Bayesian Elastic Net method
Source: BMC Genomics. 2017 Oct 16;18(Suppl 7):756. doi: 10.1186/s12864-017-4130-7 (PMC5657052; doi:10.1186/s12864-017-4130-7)
Supplement: Supplementary file 1 — Gene Ontology Enrichment Analysis. (PDF 69 kb) [file 12864_2017_4130_MOESM1_ESM.pdf]

Supplementary Table 1. Gene Ontology (GO) Enrichment Analysis Results.

	GO biological process complete	Homo sapiens - REFLIST (20814)	upload_1 (22)	upload_1 (expected)	upload_1 (fold Enrichment)	upload_1 (P-value)	-log (p-value)
Biological process	regulation of cell proliferation (GO:0042127)	1501	15	1.59	9.45	6.40E-09	8.19
	negative regulation of cell differentiation (GO:0045596)	599	11	0.63	17.37	4.87E-08	7.31
	positive regulation of cell proliferation (GO:0008284)	826	12	0.87	13.74	5.61E-08	7.25
	positive regulation of cellular process (GO:0048522)	4728	20	5	4	1.57E-07	6.8
	positive regulation of macromolecule metabolic process (GO:0010604)	2891	17	3.06	5.56	2.87E-07	6.54
	generation of neurons (GO:0048699)	1629	14	1.72	8.13	4.66E-07	6.33
	cell surface receptor signaling pathway (GO:0007166)	2470	16	2.61	6.13	4.68E-07	6.33
	negative regulation of cellular process (GO:0048523)	4246	19	4.49	4.23	5.09E-07	6.29
	tissue morphogenesis (GO:0048729)	556	10	0.59	17.02	7.35E-07	6.13
	regulation of multicellular organismal development (GO:2000026)	1698	14	1.79	7.8	8.10E-07	6.09
	neurogenesis (GO:0022008)	1707	14	1.8	7.76	8.70E-07	6.06
	negative regulation of developmental process (GO:0051093)	799	11	0.84	13.03	1.05E-06	5.98
	morphogenesis of an epithelium (GO:0002009)	419	9	0.44	20.32	1.76E-06	5.75
	regulation of developmental process (GO:0050793)	2236	15	2.36	6.35	1.96E-06	5.71
	negative regulation of biological process (GO:0048519)	4624	19	4.89	3.89	2.41E-06	5.62
	positive regulation of biological process (GO:0048518)	5476	20	5.79	3.46	2.70E-06	5.57
	regulation of cell differentiation (GO:0045595)	1492	13	1.58	8.24	2.92E-06	5.53
	epithelial tube morphogenesis (GO:0060562)	296	8	0.31	25.57	3.69E-06	5.43
	cellular response to stimulus (GO:0051716)	6613	21	6.99	3	4.41E-06	5.36
	anatomical structure morphogenesis (GO:0009653)	2415	15	2.55	5.88	5.85E-06	5.23
	positive regulation of metabolic process (GO:0009893)	3595	17	3.8	4.47	9.68E-06	5.01
	tube morphogenesis (GO:0035239)	335	8	0.35	22.59	9.71E-06	5.01
	positive regulation of gene expression (GO:0010628)	1730	13	1.83	7.11	1.80E-05	4.74
	signal transduction (GO:0007165)	5174	19	5.47	3.47	1.85E-05	4.73
	regulation of multicellular organismal process (GO:0051239)	2631	15	2.78	5.39	1.96E-05	4.71
	cell development (GO:0048468)	1742	13	1.84	7.06	1.96E-05	4.71
	tube development (GO:0035295)	564	9	0.6	15.1	2.35E-05	4.63
	cell morphogenesis involved in differentiation (GO:0000904)	854	10	0.9	11.08	4.58E-05	4.34
	positive regulation of developmental process (GO:0051094)	1150	11	1.22	9.05	4.82E-05	4.32

nervous system development (GO:0007399)	2355	14	2.49	5.62	6.10E-05	4.21
cell differentiation (GO:0030154)	3423	16	3.62	4.42	6.44E-05	4.19
single organism signaling (GO:0044700)	5561	19	5.88	3.23	6.79E-05	4.17
signaling (GO:0023052)	5564	19	5.88	3.23	6.86E-05	4.16
neuron differentiation (GO:0030182)	1208	11	1.28	8.62	8.04E-05	4.09
positive regulation of cellular metabolic process (GO:0031325)	2928	15	3.09	4.85	8.76E-05	4.06
cell communication (GO:0007154)	5650	19	5.97	3.18	9.04E-05	4.04
positive regulation of response to stimulus (GO:0048584)	2027	13	2.14	6.07	1.24E-04	3.91
nephron tubule development (GO:0072080)	75	5	0.08	63.07	1.25E-04	3.9
epithelium development (GO:0060429)	969	10	1.02	9.76	1.52E-04	3.82
renal tubule development (GO:0061326)	78	5	0.08	60.65	1.52E-04	3.82
cellular developmental process (GO:0048869)	3635	16	3.84	4.16	1.57E-04	3.8
cell morphogenesis involved in neuron differentiation (GO:0048667)	716	9	0.76	11.89	1.84E-04	3.74
neuron development (GO:0048666)	1024	10	1.08	9.24	2.56E-04	3.59
response to stimulus (GO:0050896)	8117	21	8.58	2.45	2.94E-04	3.53
regulation of response to stimulus (GO:0048583)	3823	16	4.04	3.96	3.32E-04	3.48
nephron epithelium development (GO:0072009)	93	5	0.1	50.87	3.63E-04	3.44
regulation of cell aging (GO:0090342)	33	4	0.03	> 100	3.73E-04	3.43
stem cell differentiation (GO:0048863)	196	6	0.21	28.96	3.77E-04	3.42
positive regulation of multicellular organismal process (GO:0051240)	1416	11	1.5	7.35	4.15E-04	3.38
regulation of signal transduction (GO:0009966)	2740	14	2.9	4.83	4.35E-04	3.36
positive regulation of nucleic acid-templated transcription (GO:1903508)	1428	11	1.51	7.29	4.52E-04	3.34
positive regulation of transcription, DNA-templated (GO:0045893)	1428	11	1.51	7.29	4.52E-04	3.34
response to external stimulus (GO:0009605)	2261	13	2.39	5.44	4.63E-04	3.33
positive regulation of RNA biosynthetic process (GO:1902680)	1456	11	1.54	7.15	5.52E-04	3.26
regulation of molecular function (GO:0065009)	2832	14	2.99	4.68	6.65E-04	3.18
animal organ development (GO:0048513)	2833	14	2.99	4.68	6.68E-04	3.18
positive regulation of RNA metabolic process (GO:0051254)	1497	11	1.58	6.95	7.34E-04	3.13
multicellular organism development (GO:0007275)	4737	17	5.01	3.4	7.66E-04	3.12
negative regulation of apoptotic process (GO:0043066)	855	9	0.9	9.96	8.39E-04	3.08
cell morphogenesis (GO:0000902)	1164	10	1.23	8.13	8.55E-04	3.07

regulation of localization (GO:0032879)	2390	13	2.53	5.15	9.00E-04	3.05
negative regulation of programmed cell death (GO:0043069)	865	9	0.91	9.84	9.26E-04	3.03
single-multicellular organism process (GO:0044707)	5587	18	5.91	3.05	9.79E-04	3.01
nephron development (GO:0072006)	115	5	0.12	41.13	1.03E-03	2.99
cell fate commitment (GO:0045165)	235	6	0.25	24.16	1.09E-03	2.96
system development (GO:0048731)	4147	16	4.38	3.65	1.10E-03	2.96
tissue development (GO:0009888)	1562	11	1.65	6.66	1.13E-03	2.95
columnar/cuboidal epithelial cell differentiation (GO:0002065)	118	5	0.12	40.09	1.17E-03	2.93
regulation of cell death (GO:0010941)	1574	11	1.66	6.61	1.22E-03	2.91
multicellular organismal process (GO:0032501)	6615	19	6.99	2.72	1.51E-03	2.82
cellular component morphogenesis (GO:0032989)	1240	10	1.31	7.63	1.54E-03	2.81
kidney epithelium development (GO:0072073)	125	5	0.13	37.84	1.56E-03	2.81
response to virus (GO:0009615)	251	6	0.27	22.62	1.60E-03	2.8
negative regulation of cell death (GO:0060548)	928	9	0.98	9.18	1.68E-03	2.77
glomerulus development (GO:0032835)	49	4	0.05	77.23	1.79E-03	2.75
regulation of neurogenesis (GO:0050767)	669	8	0.71	11.31	2.00E-03	2.7
regulation of signaling (GO:0023051)	3089	14	3.27	4.29	2.02E-03	2.69
branching morphogenesis of an epithelial tube (GO:0048754)	132	5	0.14	35.84	2.04E-03	2.69
axonogenesis (GO:0007409)	672	8	0.71	11.26	2.07E-03	2.68
positive regulation of macromolecule biosynthetic process (GO:0010557)	1668	11	1.76	6.24	2.21E-03	2.66
brain development (GO:0007420)	682	8	0.72	11.1	2.32E-03	2.63
regulation of cell communication (GO:0010646)	3127	14	3.31	4.24	2.36E-03	2.63
regulation of cellular process (GO:0050794)	10542	22	11.14	1.97	2.61E-03	2.58
mesenchymal cell differentiation (GO:0048762)	139	5	0.15	34.03	2.63E-03	2.58
negative regulation of multicellular organismal process (GO:0051241)	981	9	1.04	8.68	2.69E-03	2.57
axon development (GO:0061564)	697	8	0.74	10.86	2.74E-03	2.56
positive regulation of nucleobase-containing compound metabolic process (GO:0045935)	1725	11	1.82	6.03	3.10E-03	2.51
regulation of primary metabolic process (GO:0080090)	5997	18	6.34	2.84	3.17E-03	2.5
regulation of macromolecule metabolic process (GO:0060255)	6005	18	6.35	2.84	3.24E-03	2.49
regulation of metabolic process (GO:0019222)	6911	19	7.3	2.6	3.26E-03	2.49
anatomical structure development (GO:0048856)	5200	17	5.5	3.09	3.27E-03	2.49

positive regulation of transcription from RNA polymerase II promoter (GO:0045944)	1010	9	1.07	8.43	3.43E-03	2.46
head development (GO:0060322)	720	8	0.76	10.51	3.50E-03	2.46
leukocyte differentiation (GO:0002521)	293	6	0.31	19.37	3.95E-03	2.4
regulation of developmental growth (GO:0048638)	293	6	0.31	19.37	3.95E-03	2.4
regulation of cellular metabolic process (GO:0031323)	6100	18	6.45	2.79	4.19E-03	2.38
regulation of protein modification process (GO:0031399)	1781	11	1.88	5.84	4.28E-03	2.37
cell division (GO:0051301)	496	7	0.52	13.35	4.48E-03	2.35
neuron projection morphogenesis (GO:0048812)	745	8	0.79	10.16	4.53E-03	2.34
positive regulation of cellular biosynthetic process (GO:0031328)	1796	11	1.9	5.79	4.66E-03	2.33
positive regulation of developmental growth (GO:0048639)	157	5	0.17	30.13	4.77E-03	2.32
negative regulation of cell development (GO:0010721)	304	6	0.32	18.67	4.89E-03	2.31
regulation of nervous system development (GO:0051960)	755	8	0.8	10.02	5.00E-03	2.3
morphogenesis of a branching epithelium (GO:0061138)	160	5	0.17	29.57	5.23E-03	2.28
positive regulation of nitrogen compound metabolic process (GO:0051173)	1818	11	1.92	5.72	5.26E-03	2.28
positive regulation of biosynthetic process (GO:0009891)	1827	11	1.93	5.7	5.53E-03	2.26
hemopoiesis (GO:0030097)	515	7	0.54	12.86	5.76E-03	2.24
single-organism developmental process (GO:0044767)	5414	17	5.72	2.97	6.08E-03	2.22
negative regulation of ossification (GO:0030279)	67	4	0.07	56.48	6.19E-03	2.21
neuroepithelial cell differentiation (GO:0060563)	67	4	0.07	56.48	6.19E-03	2.21
positive regulation of signal transduction (GO:0009967)	1452	10	1.53	6.52	6.65E-03	2.18
negative regulation of cell aging (GO:0090344)	17	3	0.02	> 100	6.84E-03	2.16
morphogenesis of a branching structure (GO:0001763)	171	5	0.18	27.66	7.24E-03	2.14
negative regulation of neuron death (GO:1901215)	171	5	0.18	27.66	7.24E-03	2.14
regulation of apoptotic process (GO:0042981)	1470	10	1.55	6.44	7.45E-03	2.13
developmental process (GO:0032502)	5502	17	5.82	2.92	7.79E-03	2.11
regulation of biological process (GO:0050789)	11094	22	11.73	1.88	8.03E-03	2.1
morphogenesis of an endothelium (GO:0003159)	18	3	0.02	> 100	8.12E-03	2.09
regulation of programmed cell death (GO:0043067)	1485	10	1.57	6.37	8.18E-03	2.09
regulation of ossification (GO:0030278)	176	5	0.19	26.88	8.33E-03	2.08
regulation of neuron differentiation (GO:0045664)	555	7	0.59	11.93	9.47E-03	2.02
core promoter binding (GO:0001047)	153	6	0.16	37.1	2.88E-05	4.54

Molecular function	protein domain specific binding (GO:0019904)	610	8	0.64	12.41	3.26E-04	3.49
	transcription factor binding (GO:0008134)	547	7	0.58	12.11	2.83E-03	2.55
	transcription regulatory region DNA binding (GO:0044212)	803	8	0.85	9.43	2.61E-03	2.58
	regulatory region DNA binding (GO:0000975)	807	8	0.85	9.38	2.71E-03	2.57
	regulatory region nucleic acid binding (GO:0001067)	808	8	0.85	9.37	2.74E-03	2.56
	sequence-specific DNA binding (GO:0043565)	1143	8	1.21	6.62	3.57E-02	1.45
	protein binding (GO:0005515)	10761	22	11.37	1.93	1.35E-03	2.87

The first column denotes two categories including biological processes and molecular functions. The second column denotes the pathways and larger processes in biological processes and molecular functions (GO term). The header of the third column shows the entire background genes number, that is 20814 in GO database, and other values in the third column show the number of human genes (all genes in database) enriched in each GO term. The header of the fourth column shows that 22 target genes match the GO database and are used for the enrichment analysis, and the other number in the fourth column denotes the number of target genes enriched in each GO term. The fifth column shows the expected sizes for each GO term which is calculated according to the real number of enriched target genes for each GO term and the ratio of the number of human genes enriched in each GO term and all human genes in GO database. The sixth column shows the fold changes calculated as the ratio between the observed number of target genes in each GO term and the expected size from the fifth column. The seventh column shows the *p*-values calculated from the probability or chance of seeing the number of target genes out of the total genes in the list annotated to a particular GO term. The eighth column shows the –log(*p*-value).
